# BD Sports-10: A comprehensive video dataset for Bangladeshi sports classification and analysis

**DOI:** 10.1016/j.dib.2025.112442

**Published:** 2026-01-05

**Authors:** Wazih Ullah Tanzim, Niloy Barua Supta, Shifatun Nur Shifa, Khondaker A. Mamun

**Affiliations:** a Department of Computer Science and Engineering, Premier University, Academic Building #4, 44, Hazari Lane, Kotwali, Chattogram 4000, Bangladesh; b Advanced Intelligent Multidisciplinary Systems (AIMS) Lab, Institute of Research Innovation, Incubation and Commercialization (IRIIC), United International University, United City, Madani Avenue, Badda, Dhaka 1212, Bangladesh; c Department of Computer Science & Engineering, United International University, United City, Madani Avenue, Badda, Dhaka 1212, Bangladesh

**Keywords:** Bangladeshi sports classification, Video classification, Computer vision, Deep learning

## Abstract

Bangladesh has diverse and vibrant cultural sports, some of which have gained international recognition in recent years. However, there is a lack of standardized datasets for deep learning and computer vision tasks. To address this gap, BD Sports-10 was developed as a comprehensive dataset for Bangladeshi sports. It consists of ten unique sports categories, with a total of 3000 videos, 300 per class, with a resolution of 1920×1080 pixels and 30 frames per second (FPS). Each sport in the dataset features distinct rules, viewing angles, playground setups, and actions, which also depend on players’ skills. The dataset captures a diverse range of actions, including jumping, running, tagging, throwing, attempting to hit a clay pot, and capturing an opponent before they cross a designated line. BD Sports-10 includes ten traditional and culturally significant sports: *Kabaddi, Nouka Baich, Lathi Khela, Kho Kho, Kanamachi, Toilakto Kolagach Arohon (Kolagach), Hari Vanga, Morog Lorai, Lathim,* and *Joldanga*. This standardized and balanced dataset is not only suitable for classification tasks but also for object detection, player tracking, and automated scoring systems. The dataset supports research in deep learning, machine learning, and computer vision by providing ready-to-use scripts, datasets, and preprocessing pipelines that facilitate diverse AI-based experimental workflows.

Specifications Table

**Table:** 

Subject	Computer Vision and Pattern Recognition, Computer Science Applications
Specific subject area	A high-quality Bangladeshi sports video dataset designed to support deep learning (DL) applications, facilitating the classification of sports across diverse actions, perspectives, and conditions.
Type of data	Videos (.mp4) and Annotations (.xml)
Data collection	A The data were collected from popular Bangladeshi YouTube channels that showcase Bangladeshi cultural sports and games. No personal information was used, ensuring compliance with privacy concerns, and any sensitive clips were removed. To maintain consistency in temporal features, we refined the videos by eliminating sudden camera transitions. The videos were then segmented into 5-second clips, and audio was removed. Additionally, as the videos varied in lighting conditions and color settings, we adjusted brightness, sharpness, and color grading to ensure uniformity. Adobe Premiere Pro 2025 was used for these tasks, and the final videos were exported in 1920×1080 resolution using Adobe Media Encoder 2025. The videos were then anonymized using the Multi-task Cascaded Convolutional Networks (MTCNN) model.
Data source location	Videos sourced from well-known, publicly available YouTube channels documenting Bangladeshi sports, primarily filmed across various regions of Bangladesh.
Data accessibility	Repository name: BD Sports-10 Dataset (1920×1080 pixels) Data identification number: 10.57760/sciencedb.24216 Direct URL to data: https://www.scidb.cn/en/detail?dataSetId=d1e0253284cc4437811357c06f8b904b A smaller, resized version (224 × 224 pixels) is also available from Mendeley Data. Data identification number: 10.17632/rnh3x48nfb.2 Direct URL to data: https://data.mendeley.com/datasets/rnh3×48nfb/2 The dataset is publicly accessible using the provided direct URL and DOI.
Related research article	None

## 1 Value of the Data

• The BD Sports-10 dataset is the first large-scale collection of Bangladeshi indigenous sports videos, addressing the absence of high-quality, standard datasets from this domain for deep learning and computer vision research.
• It comprises 3,000 videos (300 per class across 10 sports), each acquired at 1920×1080 resolution, 30 FPS, and 5 seconds in length, ensuring temporal consistency and balanced representation across all classes.
• Each video contains 150 uniformly sampled frames, enabling fair frame-level analysis and unbiased training of deep learning models.
• The dataset includes bounding box annotations in XML format, supporting applications such as object detection, player tracking, and action recognition tasks.
• The BD Sports-10 dataset can be applied in multiple research domains, including action recognition in sports (e.g., detecting specific activities), human motion analysis (pose estimation, biomechanics, injury prevention), sports analytics (player tracking, tactical evaluation, automated scoring systems), computer vision benchmarking (testing model robustness in unconstrained environments), and edge AI research (evaluating lightweight models trained on the resized dataset for efficiency–accuracy trade-offs).
• A smaller, resized version of the dataset is also publicly available, facilitating reuse by researchers with limited computational resources. The videos are minimally edited to preserve authentic cultural and traditional characteristics, making the dataset valuable for both technical research and cultural heritage studies.

## 2 Background

High-quality, meticulously annotated image and video datasets play a vital role in advancing deep learning (DL) and computer vision. They are essential in domains such as human action recognition, motion analysis, pose estimation, and sports classification. These datasets serve as critical benchmarks for developing and evaluating algorithms capable of understanding spatial and temporal dynamics in human activities.

Early datasets such as UCF Sports [1] introduced realistic yet structured sports actions collected from BBC and ESPN broadcast footage. This dataset, comprising 150 videos with a resolution of 720×480 pixels, provided a foundation for early action recognition research. HMDB51 [2] expanded this concept by including 6,676 clips sourced from movies, public databases, YouTube, and Google, capturing diverse human facial and body actions. Later, UCF-50 [3] and UCF-101 [4] further increased both diversity and complexity, containing 50 and 101 human action classes, respectively, collected from YouTube under real-world, unconstrained conditions. Collectively, these datasets established robust benchmarks for action classification and paved the way for DL-based video analysis.

With the emergence of deep neural networks, larger and more complex datasets became necessary to model long-term temporal dependencies. Sports1M [5], containing 1,133,158 YouTube sports videos across 487 categories, marked a milestone in large-scale video learning. ActivityNet [6] followed with untrimmed real-world videos depicting a wide range of daily and recreational activities, supporting advanced research areas such as temporal action localization and activity segmentation.

For tasks requiring detailed motion understanding, fine-grained datasets such as Diving48 [7] provide high-quality clips from professional diving competitions to study precise and repetitive human movements. This dataset includes 18,404 videos across 48 fine-grained classes. FineGym [8] extended this idea with a hierarchical structure for gymnastics, capturing both event-level and element-level details for multi-level action reasoning. HAA500 [9] introduced 500 atomic human actions with clean and diverse examples, while FineDiving [10] contributed richly annotated diving videos with action types, sub-actions, temporal boundaries, and scoring information. Together, these datasets support high-resolution and fine-grained analysis of structured athletic movements.

Sports-102 [11], also known as the SYD dataset, introduced 13,593 fine-grained images capturing complex sports, yoga, and dance postures. These high-quality images facilitate research in pose estimation, activity classification, and cross-domain transfer learning within sports and fitness contexts, although the dataset lacks temporal information. While a few datasets are standardized in terms of resolution, frame rate, and class balance, most remain inconsistently organized, introducing potential bias in DL and machine learning (ML) models. Moreover, existing datasets primarily focus on international or televised sports, leaving a significant gap in regional and culturally diverse representations.

Bangladesh has a rich heritage of traditional and cultural sports, many of which remain underrepresented in digital archives. As various sports are popular in different regions, collecting video footage at the appropriate time and quality can be challenging. YouTube serves as a valuable platform for discovering and curating these cultural games. Some, such as *Kho Kho* and *Kabaddi*, have achieved international recognition, while many others remain relatively unexplored.

The InceptB dataset [12] was developed for classifying traditional Bangladeshi games and comprises 3,600 images across five categories*: Kabaddi, Danguli, Nouka Baich, Kanamachi,* and *Lathi Khela*. However, this dataset lacks temporal information and high-resolution imagery, limiting its applicability for video-based action recognition. The Traditional Bangladeshi Sports Video (TBSV) dataset [13] consists of 500 short clips (5 seconds each) recorded at 30 FPS and 1280×720 resolution, spanning five sports: *Boli Khela, Lathi Khela,* Kho *Kho, Kabaddi,* and *Nouka Baich*. However, the small sample size and limited resolution restrict its ability to represent fine-grained variations across categories. Our BD Sports-10 dataset [14] provides high-quality, large-scale, standardized videos of Bangladeshi indigenous and traditional sports. Featuring well-curated videos, the dataset preserves cultural heritage and serves as a benchmark for DL-based research in sports recognition and indigenous game analysis.

Table 1, Table 2 summarize publicly available sports and action recognition datasets and provide a comparison of their key features.

**Table 1:** Summary of publicly available sports and action recognition datasets.

Dataset Name	Year	Samples	Data Type	Classes	Frame Rate	File Format	Resolution	Background	Camera Motion	Notes
UCF Sports [1]	2008	150	Videos	10	10	.avi	720×480 pixels	Dynamic	Yes	Collected from broadcast television (BBC, ESPN) sports footage.
HMDB51 [2]	2011	6,766	Videos	51	30	Extracted Frames (.jpg)	240 pixels height (width scaled to maintain aspect ratio)	Dynamic	Yes	Collected from multiple online platforms, including movies, public repositories, YouTube, and Google, featuring distinct human actions classified into five categories: basic facial actions, facial actions involving objects, general body motions, and movements related to human interaction.
UCF-50 [3]	2012	6,676	Videos	50	Varies	.avi	Varies	Dynamic	Yes	Collected from YouTube to study 50 human action categories under unconstrained conditions.
UCF-101 [4]	2012	13,320	Videos	101	25	.avi	320×240 pixels	Dynamic	Yes	Obtained from YouTube, consisting of trimmed action clips maintained at a consistent frame rate.
Sports1M [5]	2014	1,133,158	Videos	487	Varies	Varies	Varies	Dynamic	Yes	Large-scale sports video dataset from YouTube.
ActivityNet [6]	2015	27,801	Videos	203	Varies	Varies	Varies (50% videos are 1280×720 pixels)	Dynamic	Yes	Untrimmed real-world videos of diverse human activities, sourced from Internet and YouTube.
Diving48 [7]	2018	18,404	Videos	48	Varies	.mp4	Varies	Dynamic	Yes	Video clips from diving competitions form a fine-grained diving dataset.

**Table 2:** Summary of publicly available sports and action recognition datasets (continued).

Dataset Name	Year	Samples	Data Type	Classes	Frame Rate	File Format	Resolution	Background	Camera Motion	Notes
FineGym [8]	2020	32,697	Videos	Event Level-10, Element Level-530	Varies	Undefined	Varies (95% are 720 or 1080 pixels)	Dynamic	Yes	Collected from the internet, this dataset provides fine-grained, high-quality hierarchical gymnastics actions.
HAA500 [9]	2021	10,000	Videos	500	Varies	Varies	Varies	Dynamic	Yes	Sourced from YouTube, this dataset contains fine-grained, clean, scalable, and diverse atomic human actions.
FineDiving [10]	2022	3,000	Videos	52	Varies	Varies	Varies	Dynamic	Yes	Diving events from YouTube videos of major international competitions form a fine-grained dataset with diverse actions and comprehensive annotations, including action types, sub-actions, temporal boundaries, and scores.
Sports-102 [11]	2023	13,593	Images	102	N/A	.jpg	Varies	No	No	Sourced from Kaggle and multiple websites, this dataset presents fine-grained images capturing intricate sports, yoga, and dance postures.
InceptB [12]	2018	3,600	Images	5	N/A	.jpg	Undefined	No	No	First image dataset on Bangladeshi traditional sports, collected from public sources.
TBSV [13]	2021	500	Videos	5	30	Undefined	1280×720 pixels	Dynamic	Yes	First video dataset on Bangladeshi traditional sports, collected from YouTube.
BD Sports-10 [14]	2025	3,000	Videos	10	30	.mp4	1920×1080 pixels	Dynamic	Yes	Meticulously curated from Bangladeshi sports videos on YouTube, this large-scale dataset offers high-quality videos with standardized formats, uniform resolution, and consistent temporal length of 5 seconds, ensuring reliable and reproducible research.

## 3 Data Description

**Fig. 1:**
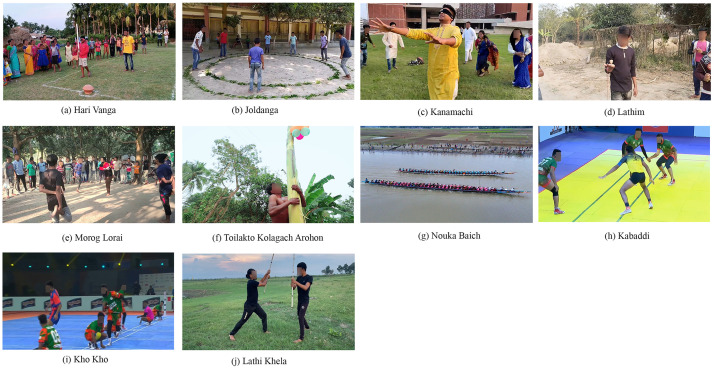
Bangladeshi Sports classes: (a) Hari Vanga, (b) Joldanga, (c) Kanamachi, (d) Lathim, (e) Morog Lorai, (f) Toilakto Kolagach Arohon (Kolagach), (g) Nouka Baich, (h) Kabaddi, (i) Kho Kho, and (j) Lathi Khela.

Fig. 1 illustrates the ten Bangladeshi sports classes in the BD Sports-10 dataset. The images represent various traditional and cultural sports that have historical connections with Bengal, and thus Bangladesh, from a thousand years ago to the present. These samples highlight variations in viewing angles, actions, and environmental conditions present in the dataset. *Hari Vanga* is a game often played in villages, where participants are blindfolded with a soft cloth and must break the *Terracotta Pot. Lathi Khela* is a very traditional game, and the practitioner is called *Lathiyal*. This game also originated in Bangladesh. Generally, two participants dance and play with a stick and shield made of bamboo and wood. In *Joldanga*, the game is played with 10 to 15 participants who must jump inside and outside, following the commands *Jol* and *Danga*. The last player who does not break the rules becomes the winner. *Kanamachi* is a famous game in both villages and towns. The blindfolded person, called *Kanamachi*, must capture one person, and that person becomes the new *Kanamachi*. It is very popular among young boys and girls. The *Lathim* game is also popular in villages, where participants tie a rope with *Lathim* and throw it on the ground to make it spin as long as possible. There are many specialized techniques to play *Lathim*, which are also included in this dataset. *Kabaddi* is the national sport of Bangladesh and is highly competitive. Though there are many rules, in simple terms, there are two teams in Kabaddi: one is the attacker and the other is the defender. From the attacking team, one player, known as the *raider*, must enter the defender’s court. The *raider* must tag as many players from the opponent’s team as possible and return to his court successfully. He scores points for every player tagged. However, if the defender’s team captures him before he touches the middle line of the court, they earn a point. The roles of the teams reverse after each turn. *Morog Lorai* is another very popular game. It is played inside a large circle, where participants must jump on one leg while holding the other leg with their hand and push one another. If any player falls to the ground, stands on two legs, or steps out of the circle, they are disqualified. As Bangladesh is a riverine country, *Nouka Baich* is very famous. This sport is highly competitive, with teams bringing their long wooden boats to a designated place on the river to compete. The boat is steered by the rowers, and there can be 60 to 70 rowers in one boat. The rowers sit in rows on both sides, singing *Sari* songs and chanting, which motivates them to increase the speed of the *Nouka. Tailakto Kolagach Arohon (Kolagach)* is a famous game in the villages of Bangladesh. In this game, a player must climb to the top of a slippery banana tree, which is coated with oil, and bring down the prize. In *Kho Kho*, a match consists of two innings, with each team having seven minutes for chasing and seven minutes for defending. The chasing team’s eight members sit alternately in the central lane’s eight squares, while the ninth member acts as the active chaser. The chaser starts from either post and *knocks out* a defender by touching them with the palm. Defenders, or runners, try to avoid being touched and must stay within the boundaries of the field. Runners enter the chase area in batches of three, and as the third runner exits, the next batch enters. Runners are considered *out* if touched by the chaser, if they step outside the boundaries, or if they enter the rectangle late. The chaser can tag any teammate sitting in the central squares to take over the chase by tapping them on the back and saying *Kho*.

**Fig. 2:**
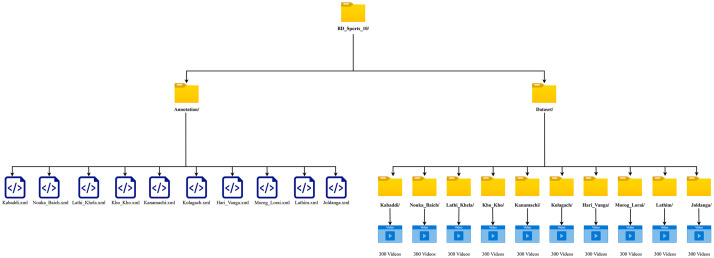
Dataset organization.

The original BD Sports-10 Dataset [14] has a size of 43.84 GB with a resolution of 1920×1080 pixels. Additionally, a resized version, BD Sports-10 Dataset (224×224 Pixels, Resized Version) [15], is available with a size of 3.51 GB. Fig. 2 presents the organization of the dataset, which is structured into two main subfolders: *Annotation* and *Dataset*. The *Annotation* folder comprises ten annotation files in .xml format, with each file corresponding to a video from one of the ten designated classes. In addition, the *Dataset* folder contains ten subfolders, each representing a distinct Bangladeshi sport, and each subfolder includes a total of 300 videos. Table 3 presents the folder structure and per-class video distribution. Table 4 presents the detailed specifications of the original BD Sports-10 dataset [14] and its 224×224 resized version [15], including the video format, codec, resolution, frame rate, and other key properties of the video files. All videos in the BD Sports-10 dataset are provided in MP4 format and encoded using H.264 / AVC / MPEG-4 AVC / MPEG-4 Part 10 or MPEG-4 Part 2. Each video has a fixed duration of 5.0 seconds and a constant frame rate of 30 frames per second, yielding 150 frames per video. Therefore, the minimum, maximum, and average video durations are all 5.0 seconds. The total duration of the dataset is approximately 4.17 hours across 3,000 videos.

**Table 3:** BD Sports-10 dataset folder structure and per-class video counts.

Sl. No.	Sports Name	Folder Name	Number of Videos
1	Hari Vanga	Hari_Vanga	300
2	Joldanga	Joldanga	300
3	Kabaddi	Kabaddi	300
4	Kanamachi	Kanamachi	300
5	Kho Kho	Kho_Kho	300
6	Toilakto Kolagach Arohon	Kolagach	300
7	Lathim	Lathim	300
8	Lathi Khela	Lathi_Khela	300
9	Morog Lorai	Morog_Lorai	300
10	Nouka Baich	Nouka_Baich	300
**Overall**			**3,000**

**Table 4:** Specifications of the BD Sports-10 dataset and its 224×224 resized version.

Sl. No.	Particulars	BD Sports-10 [14]	BD Sports-10 Dataset (224×224 Pixels, Resized Version) [15]
1	File Format	MP4	MP4
2	Video Codec	H.264 / AVC / MPEG-4 AVC / MPEG-4 Part 10 or MPEG-4 Part 2	MPEG-4 Part 2
3	Frame Rate	30 fps	30 fps
4	Frame Rate Mode	Constant	Constant
5	Resolution	1920 × 1080	224 × 224
6	Pixel Format	yuv420p	yuv420p
7	Aspect Ratio	16:9	1:1
8	Profile / Level	Main / 41 or Simple Profile / 1	Simple Profile / 1
9	Number of Frames	150	150
10	Video Bit Rate	variable	variable
11	Bit Depth	8 bits	8 bits
12	Scan Type	Progressive	Progressive
13	Duration	5 seconds	5 seconds
14	Color Representation	YUV 4:2:0	YUV 4:2:0

## 4 Experimental Design, Materials and Methods

**Fig. 3:**
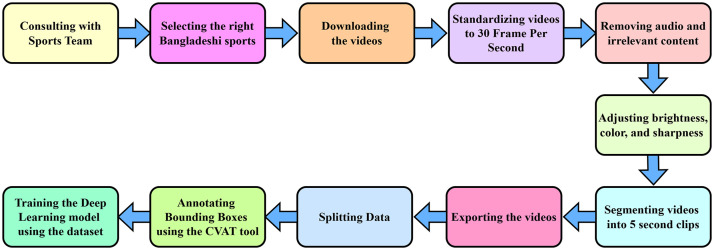
Workflow for the dataset creation.

Fig. 3 illustrates the dataset creation process. Bangladesh has diverse traditional sports across different regions. To identify which sports are deeply rooted in Bangladeshi culture, consultations were conducted with sports teams. Although many traditional sports exist, only ten Bangladeshi traditional sports were selected due to the unavailability of standardized videos meeting our requirements. The video data was collected from YouTube at a resolution of 1920×1080. Since the videos were recorded using different devices, variations in brightness, color, and sharpness were adjusted to maintain consistency, as extreme lighting conditions could introduce bias in the DL model. Although each sport has its unique characteristics, factors such as camera angles and lighting conditions varied across the dataset.

### 4.1 Raw video data preprocessing

**Fig. 4:**
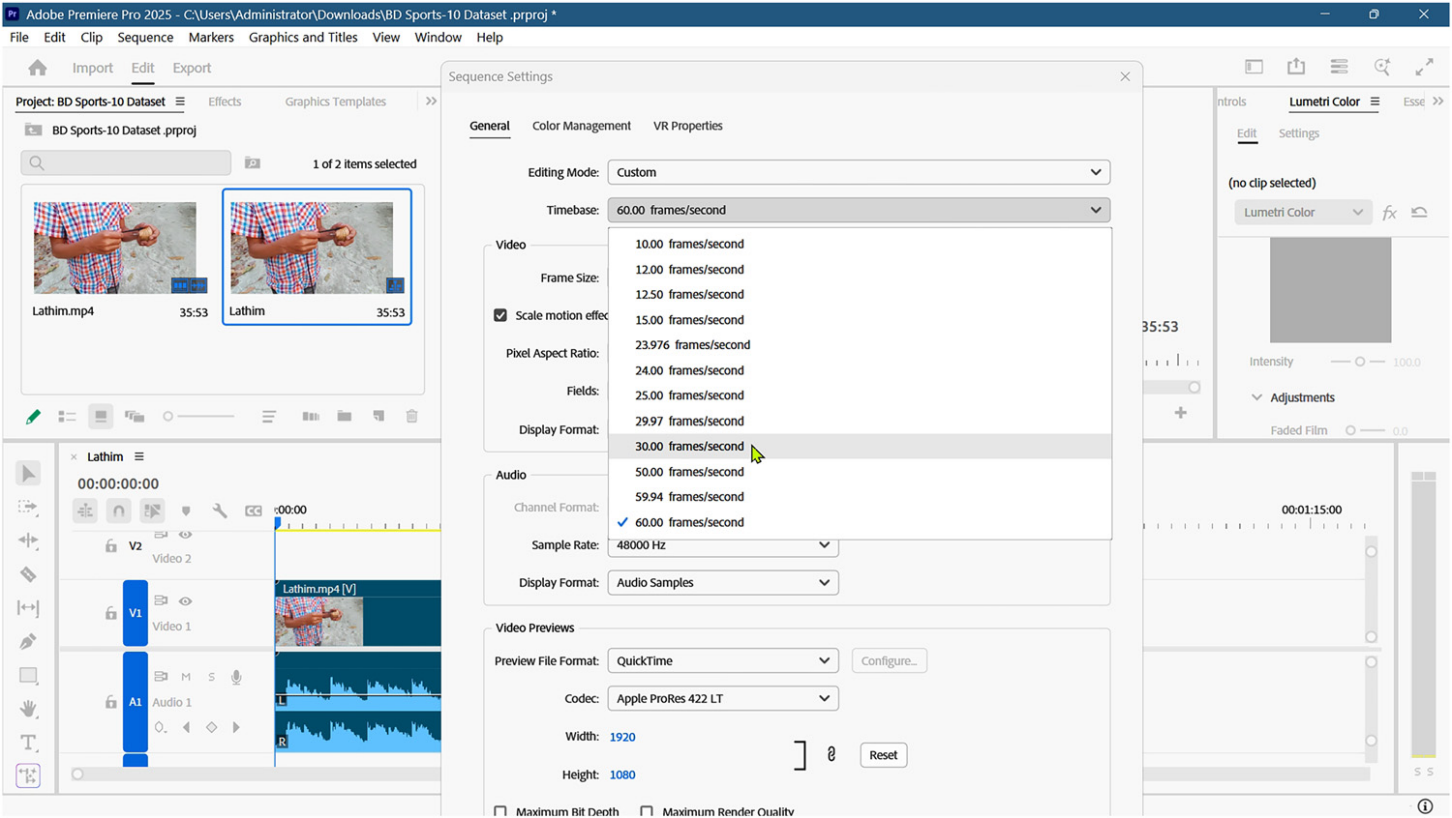
Video converted to 30 frames per second for standardization.

To preprocess raw video data into standardized 5-second clips, a systematic workflow was established using Adobe Premiere Pro 2025. The primary objective was to ensure all clips maintained a uniform resolution of 1920×1080 pixels and a consistent frame rate. Initially, a raw video (e.g., “Lathim.mp4”) and its associated audio were imported into the workspace to create a primary sequence. Normalization of frame rates was a critical step in the preprocessing phase. Since the source videos featured inconsistent frame rates—ranging from 25 to 29.97 to 60 frames per second (FPS)—all sequences were manually standardized to a constant 30 FPS. This uniformity is essential for maintaining temporal consistency across the dataset. Fig. 4 illustrates a video sequence after successful conversion to the 30 FPS standard.

Following frame rate standardization, temporal segmentation was performed to isolate relevant content. Unwanted portions of the footage were removed through precise timeline slicing, and ripple delete operations were applied to automatically eliminate any gaps created by these removals. This ensured that the resulting sequences remained temporally continuous without discontinuities in the timeline. Data cleaning also involved detaching and removing audio tracks from the video sequences. In addition to standardization, the content was filtered to remove irrelevant or sensitive information.

**Fig. 5:**
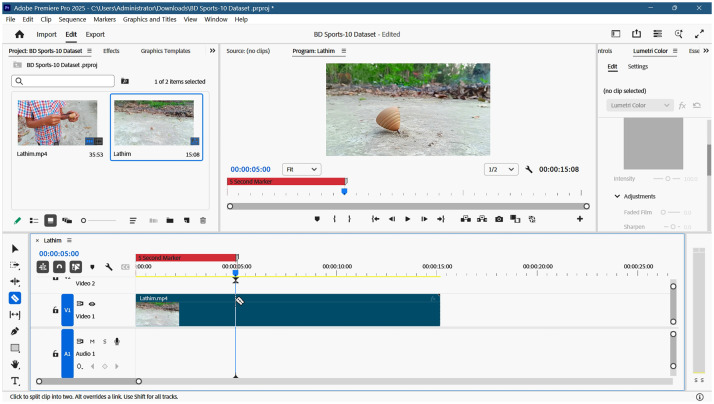
Setting a 5-second marker on the timeline to precisely trim the video sequence at 5.00 seconds using Adobe Premiere Pro.

This included excluding segments containing personal information or sudden, jarring camera transitions that could negatively impact model training or data quality. To ensure each clip met the exact requirement of a 5.00-second duration, temporal markers were utilized. By setting precise 5-second markers at the beginning of each segment and ensuring that the first video frame aligned with the zero timestamp, the clips were trimmed to an exact length. This systematic marking approach facilitated high precision during the cropping phase. Fig. 5 illustrates the application of these markers within the timeline.

Finally, the trimmed segments were organized into nested sequences to streamline the export process. These sequences were transferred to Adobe Media Encoder 2025 for batch processing and final rendering. This workflow resulted in high-resolution, standardized 5-second video files ready for dataset integration.

Detailed figures illustrating the complete preprocessing workflow are available in the data_preprocessing_trimming_and_export_figures folder of the *BD Sports-10 Dataset: Supporting Figures, Code, Notebooks, Python Package (PyPI) & Sample Data* repository archived at [16], while only selected examples are presented in this paper.

To ensure participant privacy in the BD Sports-10 dataset, an automated face anonymization pipeline was implemented using the Multi-task Cascaded Convolutional Networks (MTCNN) model [17], as provided by the facenet-pytorch library. The pipeline processes each video in a frame-by-frame manner to reliably detect and anonymize visible faces while preserving the integrity of sports actions and scene context.

Each input video is read using OpenCV, and its frame rate, spatial resolution, and total frame count are extracted to ensure that the anonymized output maintains the same temporal and spatial properties as the original video. Individual frames are converted from the OpenCV BGR color space to RGB and passed to the MTCNN face detector. The detector identifies all visible faces within a frame and returns their corresponding bounding box coordinates. The keep_all=True configuration is used to ensure that multiple faces appearing in a single frame are detected and processed.

For each detected face, the corresponding region of interest is extracted and anonymized using a Gaussian blur operation with a kernel size of 99×99 and a standard deviation of 30. These parameters were chosen to blur faces effectively without noticeably affecting the surrounding areas. The blurred face region is then reintegrated into the original frame at the same spatial location.

The anonymized frames are sequentially written to a new video file using the same frame rate and resolution as the source video, thereby preserving temporal continuity and visual consistency. All anonymized videos are saved separately from the original data to ensure ethical compliance and responsible data sharing. The complete faceblurring pipeline is implemented in the automated-face-blurring-using-MTCNN.ipynb notebook, located in the notebooks directory of the supporting repository [16].

For annotation, the Computer Vision Annotation Tool (CVAT) [18] was used, which is an open-source web-based annotation tool. First, frames were extracted from 5-second 30 FPS video clips, and 150 frames per video were manually annotated. The annotations were then exported in XML format. After completing this process, the dataset was prepared for DL model training, where frame extraction was required for model input.

**Fig. 6:**
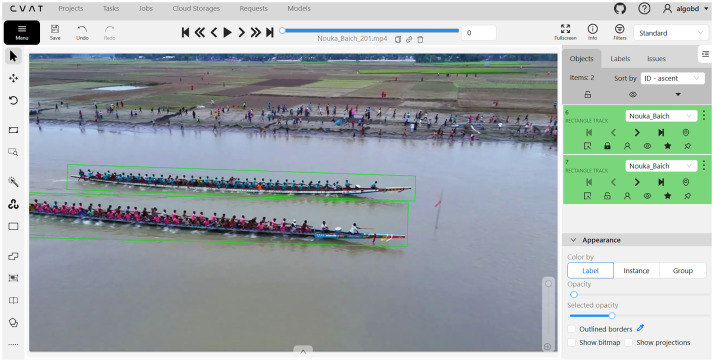
Annotated each frame of the *Nouka Baich* video using the CVAT tool.

**Fig. 7:**
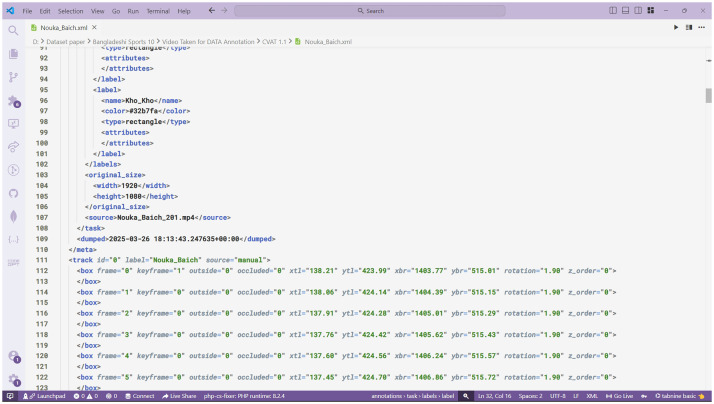
XML file generated from CVAT for video 1.1.

Fig. 6 presents an example of video frame annotation for the *Nouka Baich* class using CVAT. Each frame in the video is manually annotated to outline objects of interest, ensuring accurate labeling for training and evaluation of DL models. Fig. 7 shows the XML file generated from the *Nouka Baich* video after being exported using *CVAT for Video 1.1.*

### 4.2 Annotation format

Table 5 presents the annotation format and fields of the XML file, including the bounding box attributes. The dataset annotations are provided in CVAT XML v1.1 files (one XML file per class) and are stored in the Annotation/ directory. Each XML file contains task-level metadata, the list of label definitions, original video size, owner information, and per-frame object tracks (represented by bounding boxes). The attribute source="manual" indicates that the annotations were created manually by human annotators using CVAT, rather than automatically generated. The task uses mode=“interpolation”, where CVAT interpolates bounding boxes between labeled frames. The keyframe attribute shows whether a box is a manually labeled keyframe (1) or an interpolated frame (0).

**Table 5:** Annotation format and key XML fields of the BD Sports-10 dataset.

XML Tag	Description
<version>	CVAT XML schema version (example: 1.1).
<meta>/<task>	Task-level metadata including id, name (task name, here the class), size (number of frames), mode (e.g., interpolation), start_frame/stop_frame, creation/updated timestamps, and segment info.
<meta>/<task>/<labels>	All label definitions used by the task. Each <label> entry contains <name> (label string), <type> (annotation type, e.g., rectangle or any), and optional attributes.
<meta>/<task>/<original_size>	Original video resolution: <width> and <height> (for BD Sports-10: 1920×1080).
<meta>/<task>/<source>	Original video filename (e.g., Hari_Vanga_036.mp4).
<meta>/<task>/<owner>	The <owner> section records the annotator’s identity; for BD Sports-10, the dataset was annotated by Wazih Ullah Tanzim (username: wazih_ullah, email: wazihullahtanzim@gmail.com).
<track>	A single tracked object instance, with attributes id, label (class), and source (e.g., manual). A <track> groups a sequence of <box> elements for that object.
<box>	Frame-level bounding box with attributes: frame (frame index, starting at 0), keyframe (1 = manually labeled keyframe; 0 = interpolated), outside (1= object outside frame; 0 = visible), occluded (1 = partially/fully occluded; 0 = fully visible), xtl, ytl (top-left coordinates), xbr, ybr (bottom-right coordinates), rotation (bounding box rotation in degrees), z_order (stacking order; higher values drawn on top).

## Limitations

None

## Ethics Statement

All procedures for the collection of Bangladeshi sports videos in this dataset were conducted in compliance with the ethical guidelines and regulations of Premier University (PU), and in accordance with the principles of the Declaration of Helsinki. Ethical approval was granted by the Ethics Committee of the Department of Computer Science and Engineering (DCSE), PU (Ref. No: EC—CSE-2024–11–09, dated November 9, 2024). Informed consent was obtained from all individuals appearing in the videos prior to collection. All video data were anonymized to ensure privacy, and no personal or sensitive information was collected. The videos were strictly used for academic research and validated by domain experts. This study complies with institutional and international ethical standards for research involving human participants, and the privacy rights of all individuals have been fully respected.

## Data Availability

The BD Sports-10 Dataset is publicly available in two versions: the original high-resolution dataset and a resized version. The original dataset can be accessed via BD Sports-10 Dataset (Original data) (Science Data Bank). The resized version (224×224 pixels) is available on BD Sports-10 Dataset (224×224 Pixels, Resized Version) (Original data) (Mendeley Data). The BD Sports-10 Dataset is also provided as a Python package, which allows users to easily download and use the dataset in Python environments such as Jupyter Notebook, Kaggle, or Google Colab. The resized version (224×224 pixels) can be installed using **!pip install bd-sports-10-resized=0.6.0**, while the original full-resolution version (1920×1080 pixels) is available via **!pip install bd-sports-10-original=0.4.0.** This approach enables straightforward access to the dataset. The PyPI packages are available at: BD Sports-10 Original and BD Sports-10 Resized.

## Supplementary Materials

All scripts and notebooks utilized for the downloading, preprocessing, extraction, and analysis of the dataset are publicly accessible in the GitHub repository https://github.com/Wazih-Ullah-Tanzim/BD-Sports-10, which has been archived and is citable via Zenodo (version v1.0.0) at https://doi.org/10.5281/zenodo.17517571 [16].

## CRediT Author Statement

**Wazih Ullah Tanzim:** Conceptualization, Formal analysis, Methodology, Software, Writing - Original Draft, Visualization, Validation, Investigation, Data Curation. **Niloy Barua Supta:** Data Curation, Visualization, Validation. **Shifatun Nur Shifa:** Data Curation, Visualization. **Khondaker A. Mamun:** Conceptualization, Methodology, Supervision, Project Administration, Guidance, Writing -Review & Editing.

## Data Availability

Science Data BankBD Sports-10 Dataset (Original data). Mendeley DataBD Sports-10 Dataset (224×224 Pixels, Resized Version) (Original data).
